# Hand exercise for women with rheumatoid arthritis and decreased hand function: an exploratory randomized controlled trial

**DOI:** 10.1186/s13075-019-1924-9

**Published:** 2019-06-26

**Authors:** Karen Ellegaard, Cecilie von Bülow, Alice Røpke, Cecilie Bartholdy, Inge Skovby Hansen, Signe Rifbjerg-Madsen, Marius Henriksen, Eva Ejlersen Wæhrens

**Affiliations:** 1 The Parker Institute, Copenhagen University Hospital Bispebjerg and Frederiksberg, Nordre Fasanvej 57, DK-2000 Copenhagen F, Denmark; 2 The Research Initiative for Activity Studies and Occupational Therapy, General Practice, Department of Public Health, University of Southern Denmark, Odense, Denmark; 3 Metropolitan University College, Institute for Occupational Therapy and Physiotherapy, Copenhagen, Denmark; 4 Department of Physical and Occupational Therapy, Copenhagen University Hospital Bispebjerg and Frederiksberg, Copenhagen, Denmark

**Keywords:** Rheumatoid arthritis, Hand function, ADL performance, Exercise program, Observational based assessment

## Abstract

**Background:**

People with hand-related rheumatoid arthritis (RA) experience problems performing activities of daily living (ADL). Compensatory strategies to improve ADL ability have shown effective. Similarly, hand exercise has shown effect on pain, grip strength, and self-reported ability. A combination has shown positive effects based on self-report, but self-report and observation provide distinct information about ADL. The purpose of this study was to examine whether hand exercise as add on to compensatory intervention (CIP) will improve observed ADL ability in RA.

**Methods:**

Women (*n* = 55) with hand-related RA were randomized to CIP_EXERCISE_ (intervention) or CIP only (control). CIP is focused on joint protection, assistive devices, and alternative ways of performing AD. The hand-exercise program addressed range of motion and muscle strength.

Primary outcome was change in observed ADL motor ability measured by the Assessment of Motor and Process Skills (AMPS). Baseline measures were repeated after 8 weeks.

**Results:**

Improvements in ADL motor ability in CIP_EXERCISE_ (mean change = 0.24 logits; 95% CI = 0.09 to 0.39) and CIP_CONTROL_ (mean change =0.20 logits; 95% CI = 0.05 to 0.35) were statistically significant, with no differences between groups (mean difference = 0.04 logits; 95% CI = − 0.16 to 0.25). Thirteen (46.4%) participants in the CIP_EXERCISE_ and 12 (44.4%) in the CIP_CONTROL_ obtained clinically relevant improvements (≥ 0.30 logits) in ADL motor ability; this group difference was not significant (*z* = 0.15; *p* = 0.88).

**Conclusion:**

Adding hand exercise to a compensatory intervention did not yield additional benefits in women with hand-related RA.

The study was approved by the ethics committee 14th of April 2014 (H-3-2014-025) and registered at ClinicalTrials.gov 16th of May 2014 (NCT02140866).

**Electronic supplementary material:**

The online version of this article (10.1186/s13075-019-1924-9) contains supplementary material, which is available to authorized users.

## Introduction

Rheumatoid arthritis (RA) is a chronic autoimmune disease characterized by inflammation of synovium [1]. In 90% of RA patients, joints of the hand are affected resulting in problems performing activities of daily living (ADL) [2, 3]. A study by Thyberg et al. indicates that low grip strength may play a role in decreased ADL ability [4].

Hand-exercise programs may have positive effects on hand function in RA [5–9]. A randomized trial including patients with RA-related hand problems showed positive effects on hand function after hand exercise added to a 1.5-h instruction in joint protection, without negative effect on disease activity [8–10].

Compensatory programs based on individualized joint protection and education in coping strategies to improve performance of ADL tasks have shown to be effective [11–13]. Hammond et al. found that patients with RA, diagnosed within < 5 years, attending an educational-behavioral joint protection program, maintained ADL ability after 4 years [11, 12]. Masiero et al. showed that patients with RA presented with less pain and disability 8 months after an educational-behavioral joint protection program [13].

Clinical guidelines recommend that patients with RA receive hand-exercise programs focusing on increasing muscle strength and movements and programs on compensatory strategies to overcome difficulties in ADL task performance related to hand impairments [14].

However, existing evidence of the effectiveness of hand exercise on functional ability is based on self-reported data, typically questionnaires. While self-report represents the patient’s perspective, it is documented that measures of self-reported ADL ability have low to moderate relationship to observation-based ADL ability measures [3, 15]. Thus, self-reported and observation-based measures provide distinct but complementary information [3, 15]. Observation-based evaluations of ADL ability are considered to provide more neutral measures than evaluations based on self-report, as observations seem less influenced by psychosocial factors and pain [16]. Furthermore, observation-based evaluations have shown to be more sensitive to change after intervention among patients with chronic pain [17].

It remains to be tested whether hand-exercise therapy as an add on to a compensatory intervention program (CIP) will improve the observable performance of ADL tasks requiring hand function in patients with RA. Moreover, as suppression of disease activity is essential to avoid progression of joints destruction [18, 19], it is relevant to explore changes in disease activity after therapeutic interventions.

It was hypothesized that hand-exercise therapy as an add on to CIP in patients with RA-related hand impairments would result in larger improvements in observed ADL ability as compared to CIP alone, without increasing disease activity.

## Patients and methods

### Participants

Participants were females with RA reporting ADL task performance problems involving the hands, recruited from May 2014 to January 2016 through rheumatologists at outpatient clinics in Copenhagen and announcements in daily press. Potential participants were given further information and pre-screened for eligibility via telephone. If inclined to participate, they were examined by a rheumatologist to determine if they fulfilled inclusion criteria.

Inclusion criteria are as follows: females aged > 18 years; diagnosed with RA (ACR/EULAR 2010 Criteria) [20]; involvement of minimum of one tender wrist, MCP, or PIP joint; stable medication 3 months prior to participation, and self-reported decreased ability to perform ADL tasks involving the hands. Exclusion criteria are as follows: significant osteoarthritis of the hand (assessed by the rheumatologist); hand surgery within 6 months; other pain condition involving muscles and/or joints; prednisolone therapy; alternative treatments during study period; change in medical treatment during study period; inability to understand Danish; and finally any other contradictions for participating assessed by the rheumatologist. Participants were asked not to participate in occupational or physical therapy interventions elsewhere during the study period, aquatic physical therapy was allowed.

### Ethics approval and consent to participate

Participants gave written informed consent. The study was approved by the ethics committee of the Capital Region of Denmark (H-3-2014-025) and registered at ClinicalTrials.gov (NCT02140866). The study was conducted in accordance with standards of the responsible committee on human experimentation and the Declaration of Helsinki. If participants experienced increased disease activity during the study period, US and rheumatologist examinations were made to determine if modifications or withdrawal were needed.

### Study design and randomization

The investigator-initiated study was designed as an RCT with parallel groups. Participants entered an 8-week program and were randomly assigned to either intervention group (hand-exercise therapy and CIP, CIP_EXERCISE_) or control group (CIP alone, CIP_CONTROL_) with a 1:1 equal allocation ratio utilizing a concealment process. Randomization was made using sealed envelopes and carried out by the project secretary. The outcome assessors and data analysts were kept blinded to the allocation, and participants were instructed not to convey their group allocation. The persons performing US examination and evaluation were blinded to results of clinical examination and group allocation.

### Intervention programs

The CIP consisted of an introduction to compensatory strategies including joint protection (JP), assistive devices, and alternative methods of performing ADL [21, 22]. The program was client-centered focusing on improving the ability to perform those ADL tasks that the single participant identified as purposeful to her life. It consisted of four 1-h sessions during an 8-week period. One occupational therapist (OT) (ISH) with > 10 years of experience performed all sessions. The first session aimed to identify the participant’s individual resources and problems in ADL task performance, goal setting focused on ADL task performance, and introduction to assistive devices. As homework, assistive devices were applied at home and the utility evaluated. In the second session, the OT presented JP principles and discussed these in relation to the participant’s ADL task problems. She supervised the participant to integrate JP principles and alternative methods of doing in ADL tasks. In the third session, the OT followed up on the use of JP principles and assistive devices at home and together they identified any additional ADL task, representing a challenge, and performed this under supervision. Optionally, additional assistive devices were handed out. As homework, the participant practiced JP principles and assistive devices in the chosen task. Fourth session, the OT followed up on homework, evaluated goals, and introduced how to apply for assistive devices and housing accessibility solutions in the home municipality. This fourth session, if relevant, was done as a telephone meeting.

The hand-exercise program lasted for 8 weeks as a strength increase is possible within this time frame and was designed based on recent research [6, 8, 23–25]. First, the exercise intervention was performed four times per week with one session supervised by a physical therapist (PT), and the other three were home based. After 14 days. One PT (CB) with 3 years of experience performed the individual exercise sessions. Home-based exercise sessions were recorded in a diary. During the period, the exercise intervention was increased to once daily and the load was increased. The program consisted of three parts: (1) warm-up/mobility (10 min), (2) muscle strength training (20 min), and (3) cool-down (5 min). The warm-up was performed to prepare the joints for the muscle strength training and to improve flexibility; the muscle strength training was designed to ensure that relevant muscle was targeted within a period of 20 min. Resistance was supplied by exercise bands and Thera-putty. The amount of resistance was based on the weakest hand. The resistance intensity was set according to the participant’s self-reported experience of load using the Borg Scale [26]. This load was chosen to minimize the risk of flare-up symptoms due to overload and to allow for progression. For cool-down, some of the warm-up exercises were receded. All participants received a detailed illustrated description of the exercise program (Additional file 2). In case of any flare-up in symptoms, the participant was set to only conduct the warm-up and cool-down part. Post exercise soreness and temporary fatigue was tolerated. Detailed description of the exercise program is presented in Additional file 3. If a participant failed to meet at an appointment, a phone call was made to maintain fidelity.

### Outcomes

Primary outcome was observed, ADL motor ability measured by Assessment of Motor and Process Skills (AMPS).

Secondary outcomes were observed, ADL process ability (AMPS), self-reported ADL ability (ADL-Questionnaire, ADL-Q), self-reported disability (Stanford Health Assessment Questionnaire Disability Index, HAQ-DI), overall disease activity (DAS28), grip strength, and pain. Exploratory outcome was disease activity assessed by US.

### AMPS

The AMPS is a standardized observation-based tool used to measure a person’s observed ADL task performance [27, 28]. The person being evaluated chooses and performs at least two standardized ADL tasks of relevance and appropriate challenge. During AMPS evaluation, two domains are evaluated, i.e., ADL motor ability (the amount of effort, fatigue and/or clumsiness) and ADL process ability (the degree of disorganization, inappropriate use of time, space, objects and ability to adapt actions). The 16 ADL motor and 20 ADL process skills are evaluated in terms of ease, efficiency, safety, and independence using a four-point ordinal scale. The available AMPS software [29], based on a many-faceted Rasch measurement model, makes it possible to convert ordinal raw scores into overall linear ADL motor ability measures and overall linear ADL process ability measures adjusted for task challenge, skill item difficulty, and rater severity. Measures are expressed in logits (log-odds probability units) [28]. The overall ADL motor ability measure indicates how much effort or fatigue the person demonstrated, and the overall ADL process ability measure indicates how efficient the person was observed to be during the ADL task performance. Additionally, both ADL ability measures reflect safety and independence in ADL task performance. ADL ability measures above the 2.0 logit cutoff on the ADL motor scale and above the 1.0 logit cutoff on the ADL process scale indicate effortless, efficient, safe, and independent ADL task performance in everyday life. In contrast, ADL motor ability measures below the 2.0 logits cutoff indicate increased effort or fatigue during task performance. Moreover, ADL ability measures below the 1.50 ADL motor cutoff and/or below the 1.00 ADL process cutoff indicate a need for minimal assistance for community living. Finally, according to the AMPS manual a difference of > 0.30 logits on the AMPS ADL motor and/or ADL process scale defines a clinically relevant change [28]. Studies support that the AMPS ability measures are reliable and valid in RA patients [3].

### ADL-Q

Self-reported ADL ability was assessed using the ADL-Q, a standardized instrument with 47 ADL tasks developed to measure a person’s perceived quality of ADL performance [3, 30]. The person marks the quality of the ADL task performance using seven response categories reflecting efficiency, effort/fatigue, safety, and independence. Rasch methods are applied [30]. Studies support that the ADL-Q can be used to generate valid measures of self-reported quality of ADL task performance among RA patients [3].

### HAQ-DI

The HAQ-DI is developed to assess disability in RA [21]. The questionnaire consists of 20 questions primarily concerning ADL tasks, a Danish version was used [31].

### DAS28

Overall disease activity was assessed using the DAS28 which is based on count of 28 joints for swelling and tenderness, C-reactive protein level in the blood and the patient’s self-reported impact of disease on a visual analog scale (VAS Global health). The DAS28 score range from 0 to 9.4 [32].

### Grip strength

The maximal grip strength was measured in kilogram using a digital hand Dynamometer (North Coast Medical Inc.). The grip strength was measured three times in both hands. The maximal force performed in each hand was used in the analysis.

### Pain

Hand pain during activity and in rest was measured in both hands on a visual analog scale (VAS), where zero was no pain and 100 was maximal pain.

### Ultrasound examination

Synovial hypertrophy and increased synovial perfusion are indications of disease activity assessed by US [33]. Gray scale US is used to examine synovial hypertrophy seen as hypo-echoic structure between the tendon/muscle and the bone [1]. Doppler US added to the gray scale image register movement of the blood as an indication of increased synovial perfusion. US has shown to correlate with measures of disease activity in RA [19, 34, 35]. US examination in RA has shown to display sub clinical disease activity leading to disease progression on X-ray [36]. The wrist and MCP 2–5 were examined both dorsal and palmar. The wrist was scanned in four dorsal and one volar position and the MCP joints in three dorsal and one volar position. Both synovial hypertrophy and Doppler were evaluated using a validated scoring system for RA [37]. One score for synovial hypertrophy, one score for synovial perfusion (Doppler), and one sum score were calculated.

### Sample size

Sample size calculation was based on previous data on AMPS ADL motor ability in women with RA [3]. For a two sample pooled *t*-test of a normal mean difference with pooled variances (equal variances assumed) and a two-sided significance level of 5% (*p* ≤ 0.05), assuming a common standard deviation (SD) of 0.36 logits, a sample size of *n* = 32 participants per group was required to obtain a power of at least 90% to detect a group mean difference of 0.3 logits. It was decided to include *n* = 45 participants in each group.

### Statistical analysis

Data analyses were carried out according to a pre-established statistical analysis plan (SAP); all analyses were done applying SAS (v. 9.4, SAS Institute Inc., Cary, NC, USA). Descriptive statistics and tests are reported in accordance to the “Enhancing the QUAlity and Transparency Of health Research” (EQUATOR) network [38, 39]. To evaluate the empirical distributions of the continuous outcomes, visual inspection was applied to suggest whether the assumption of normality was reasonable. The PROCUNIVARIATE statement was used for summarizing the data (descriptive statistics).

Intention-to-treat (ITT) analyses were made, i.e., analyzing participant outcomes according to the group to which they were allocated, even if participants did not receive allocated intervention. The ITT principle was done by replacing missing data with the value obtained at baseline.

At week 8, the CIP_EXERCISE_ group was compared with the CIP_CONTROL_ group using general linear model (analyses of covariance; ANCOVA) for mean changes from baseline and *t* tests for comparison of least squares means between groups. The model included change as the dependent variable (Δ), with treatment group as a main effect and the baseline score as an additional covariate. Results were expressed as the difference between the group means and 95% CI with the associated *p* values, based on the general linear model.

For sensitivity purposes, the analyses were repeated with further adjustment for disease duration and hand pain at baseline as there were group imbalances in these variables at baseline (Additional file 1: Table S1).

The proportion of participants responding to therapy (≥ 0.30 logits on the AMPS ADL motor scale) was analyzed using *z* test to evaluate the difference in the number of responders between groups.

## Results

**Fig. 1:**
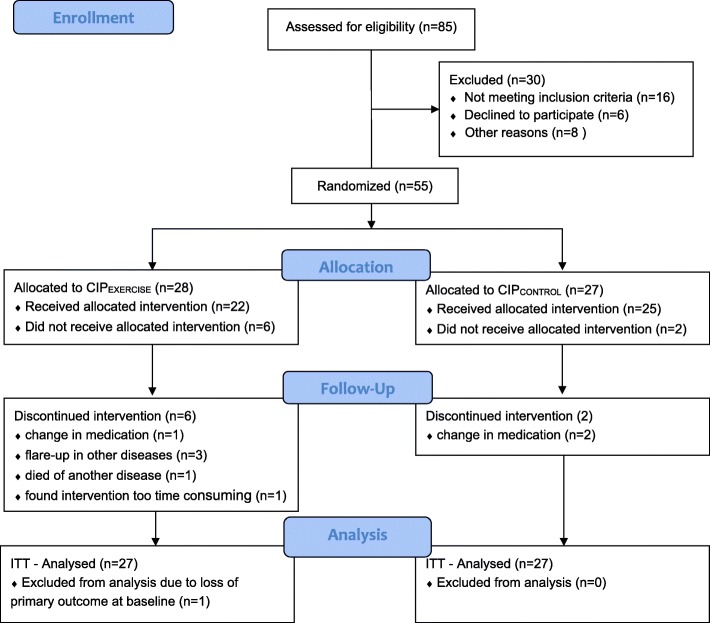
CONSORT flow diagram of the study

Fifty-five were randomized to CIP_EXERCISE_ (*n* = 28) or CIP_CONTROL_ (*n* = 27); 22 and 25 participants, respectively, completed the trial (Fig. 1). Reason for drop out in CIP_CONTROL_ was change in medication. In CIP_EXERCISE_, one changed medication, three had flare-up in other diseases, one died of another disease, and one found the intervention too time consuming. Due to loss of the AMPS ADL motor ability measure at baseline for one participant in CIP_EXERCISE_, the ITT population consisted of *n* = 27 in each group. The ITT population’s mean age was 63.7 (SD = 13.0) years, and mean disease duration was 12.6 (SD = 11.1) years. Mean baseline tender and swollen joint count (28 joints) was 5.0 (SD = 4.8) and 1.4 (SD = 1.7), respectively; the mean hand pain in activity was 42.7 (SD = 26.1) (right) and 36.2 (SD = 27.6) (left) mm VAS. Mean baseline AMPS ADL motor ability measure was 1.4 (SD = 0.5) logits. Mean baseline HAQ score was 1.1 (SD = 0.6). For other baseline characteristics, see Table 1.

**Table 1:** Baseline characteristics for all randomized participants

	CIP_EXERCISE_ (n=27) Mean (SD)/ Median (IQR)	CIP_CONTROL_ (n=27) Mean (SD)/ Median (IQR)	Total (54) Mean (SD)/ Median (IQR)
Age (Years)	64.8 (13.5)	62.6 (12.0)	63.7 (12.8)
Weight (Kg)	69.4 (12.3)	70.0 (11.9)	70.2 (12.5)
Height (Cm)	167.6 (7.4)	166.2 (7.4)	166.9 (7.4)
Start symptoms (Years)	11.3 (3.5:22.5)	14.1 (7.9:18.2)	11.9 (4.7:20.3)
Disease duration (Years)	3.1 (0.4:11.6)	10.3 (7.3:14.6)	7.6 (1.6:13.5)
AMPS ADL motor^a^ (Logits)	1.34 (0.4)	1.38 (0.5)	1.36 (0.5)
AMPS ADL process^a^ (Logits)	1.20 (0.2)	1.13 (0.2)	1.27 (0.2)
ADL-Questionnaire (Logits)	0.4 (0.1)	0.5 (0.5)	0.5 (0.3)
HAQ-DI^b^	1.1 (0.6)	1.1 (0.6)	1.1 (0.6)
Hand strength and pain			
Maximal grip strength (Kg)			
Right	17.7 (7.7)	18.7 (8.1)	18.2 (7.8)
Left	17.2 (6.3)	17.8 (5.7)	17.5 (6.0)
Hand pain: Activity (mm VAS)			
Right	50 (25.0:62.0)	32 (19.5:68.5)	40.5 (21.25:63:75)
Left	50 (11.0:65.5)	26 (10.5:52.0)	30.5 (11.0:60.25)
Hand pain: Rest (mm VAS)			
Right	29 (7.0:54.5)	19 (10.0:50.0)	22 (10.0:51.5)
Left	39 (10.0:50.0)	14 (5.5:25.5)	16.5 (6.25:42.5)
Medication: PainKillers (Number per day)	1.7 (1.2)	1.6 (1.0)	1.6 (1.1)
DAS28^c^	3.6 (1.1)	3.2 (1.1)	3.4 (1.1)
Tender joint count (28 joints)	5.8 (5.7)	4.1 (3.7)	5.0 (4.8)
Swollen joint count (28 joints)	1.5 (1.7)	1.3 (1.7)	1.4 (1.7)
CRP^d^	5.3 (6.6)	5.4 (9.8)	5.3 (8.1)
Disease activity (mm) (VAS)	47.19 (26.86)	41.19 (24.93)	44.19 (25.85)
ESR^e^	16.1 (11.3)	15.2 (12.7)	15.6 (11.9)
UltraSound (US)			
US score: synovial hypertrophy (0-126)	16.5 (15.6)	20.4 (23.2)	18.5 (19.8)
Synovial perfusion (Doppler activity) (0-126)	5.6 (11.6)	6.7 (12.8)	6.2 (12.1)
US score: sum (0-252)	22.2 (25.9)	27.1 (35.2)	24.7 (30.9)

^a^*AMPS* Assessment of Motor and Process Skills

^b^*HAQ-DI* Stanford Health Assessment Questionnaire Disability Index

^c^*DAS28* Disease Activity Score 28

^d^*CRP* C-reactive protein

^e^*ESR* Erythrocyte Sedimentation Rate

The CIP_EXERCISE_ group attended a mean of 2.4 (SD = 1.2) CIP sessions, whereas the participants in the CIP_CONTROL_ attended 2.7 (SD = 1.0) CIP sessions. The average number of hand-exercise sessions was 20.2 (SD = 10.3). Exercise diary was received from 24 of the 27 participants. The hand-exercise program was delivered as intended (see Additional file 3).

No significant mean differences in changes from baseline were seen between groups in any of the outcomes (Table 2). Still, tendencies towards differences between groups in changes in DAS28 score, CRP, and ESR were seen. Improvements in primary outcome, observed ADL motor ability, were seen in both groups, in the CIP_EXERCISE_ (ADL motor mean change = 0.24 logits; 95% CI = 0.09 to 0.39) and CIP_CONTROL_ (ADL motor mean change = 0.20 logits; 95% CI = 0.05 to 0.35), however with no statistically significant difference between groups (ADL motor mean difference = 0.04 logits; 95% CI = − 0.16 to 0.25). A significant increase in grip strength in the right hand was seen in the CIP_EXERCISE_ group and ESR increased significantly in the CIP_CONTROL_ group (Table 2).

**Table 2:** Changes from Baseline in Primary and Secondary Outcomes Analyses based on the Intention-To-Treat-Population

	CIP_EXERCISE_ (n=27) Mean change (95% CI)	CIP_CONTROL_ (n=27 ) Mean change (95% CI)	Group difference Mean change (95%CI)	P-value
AMPS^a^ ADL motor ability	0.24 (0.09 to 0.39)	0.20 (0.05 to 0.35)	0.04 (-0.16 to 0.25)	0.70
AMPS^a^ ADL process ability	0.05 (-0.05 to 0.14)	0.02 (-0.07 to 0.12)	0.02 (-0.11 to 0.16)	0.73
ADL-Questionnaire (ADL-Q)	0.09 (-0.01 to 0.20)	0.09 (-0.02 to 0.19)	0.01 (-0.15 to 0.16)	0.90
HAQ-DI^b^	-0.09 (-0.19 to 0.01)	0.01 (-0.09 to 0.11)	-0.1 (-0.24 to 0.04)	0.16
Hand strength and pain				
Max grip strength (Kg)				
Right	1.43 (0.40 to 2.45)	0.18 (-0.86 to 1.23)	1.24 (-0.22 to 2.71)	0.10
Left	1.00 (-0.47 to 2.47)	0.36 (-0.71 to 3.44)	1.36 (-0.71 to 3.44)	0.20
Hand pain, Activity (mm VAS				
Right	-1.10 (-7.90 to 5.78)	0.57 (-6.27 to 7.41)	-1.63 (-11.30 to 8.04)	0.74
Left	-5.26 (-12.80 to 2.28)	-0.78 (-8.31 to 6.76)	-4.48 (-15.28 to 6.30)	0.41
Hand pain, Rest (mm VAS)				
Right	-1.43 (-8.63 to 5.78)	2.87 (-4.33 to 10.07)	-4.30 (-14.49 to 5.00)	0.40
Left	-1.67 (-9.71 to 6.36)	4.41-(-3.62 to 12.45)	-6.08 (-17.80 to 5.62)	0.30
PainKillers (Number per day)	0.01 (-0.19 to 0.22)	0.01 (-0.22 to 0.19)	0.02 (-0.26 to 0.31)	0.87
DAS28^c^	-0.17 (-0.49 to 0.15)	0.26 (-0.10 to 0.53)	-0.39 (-0.84 to 0.07)	0.09
Tender joint count	-0.51 (-1.83 to 0.82)	0.38 (-0.93 to 1.68)	-0.88 (-2.75 to 0.99)	0.35
Swollen joint count	0.23 (-0.27 to 0.72)	0.15 (-0.33 to 0.63)	0.08 (-0.61 to 0.77)	0.82
CRP^d^	-0.67 (-2.44-1.11)	1.76 (-0.12-3.63)	-2.42 (-5.00-0.15)	0.06
Disease activity (VAS)	-7.97 (-15.86—0.09)	-0.70 (-7.93 to 6.53)	-7.27 (-17.97 to 3.43)	0.18
ESR^e^	-0.50 (-2.52 to 1.51)	2.30 (0.29 to 4.32)	-2.80 (-5.65 to 0.05)	0.05
Ultra Sound (US)				
US score: synovial hypertrophy	0.18 (-2.0 to 2.32)	1.83 (-0.22 to 3.89)	-1.66 (-4.63 to 1.32)	0.27
Synovial perfusion (Doppler)	-0.38 (-2.47 to 1.72)	1.01 (-0.99 to 3.03)	-1.39 (-4.29 to 1.51)	0.34
UL score total	-1.81 (-5.76 to 2.34)	2.56 (-1.23 to 6.36)	-4.37 (-9.86 to 1.11)	0.12

^a^*AMPS* Assessment of Motor and Process Skills

^b^*HAQ-DI* Stanford Health Assessment Questionnaire Disability Index

^c^*DAS28* Disease Activity Score 28

^d^*CRP* C-reactive protein

^e^*ESR* Erythrocyte Sedimentation Rate

^f^*AntiCCP* anti Cyclic Citrullinated Peptides

The sensitivity analyses confirmed the primary analyses and supported further the tendencies towards group differences in DAS28, HAQ-DI, and ultrasound total score (Additional file 1: Table S1).

The responder analysis revealed that 13 (46.4%) participants in the CIP_EXERCISE_ and 12 (44.4%) in the CIP_CONTROL_ obtained clinically relevant improvements (≥ 0.30) in ADL motor ability, and this difference was not significant (*z* = 0.15; *p* = 0.88).

## Discussion

Based on the results of this study, it was not possible to confirm the hypothesis that hand-exercise therapy as add on to a compensatory intervention program in patients with decreased ADL ability, following RA-related hand impairments, would result in larger improvements in observed ADL ability as compared to CIP alone.

While no statistically significant differences in changes in primary and secondary outcomes were seen between groups, both groups obtained statistically significant improvements in the primary outcome, observed ADL motor ability. While the mean increases in ADL motor ability in both groups were not clinically relevant, responder analysis revealed that almost half of the participants (46.3%) achieved a clinically relevant (i.e., > 0.3 logits) increase in observed ADL ability. These results, and the fact that the number of responders in each group was almost the same, suggest that a large percentage of the participants benefitted from the CIP intervention.

The CIP_EXERCISE_ group received hand exercise as a restorative add on to the CIP intervention. It was assumed that increasing strength and flexibility of the hands would translate into improved ADL ability. While this was not the case, participants in the CIP_EXERCISE_ group still obtained a significant increase in grip strength, which was not observed in the CIP_CONTROL_ group. This suggests that hand exercise may improve strength, but that such improvements not necessarily translate into improved ADL ability. This indication of a benefit of the exercise program is further implied by a minor decrease in DAS28 score seen in the CIP_EXERCISE_ group as compared to the CIP_CONTROL_ group. However, the change in DAS28 was only minor and is not reaching the cutoff for a clinically relevant change [40] and was not translated into improved ADL ability.

Despite statistically significant increases in observed ADL ability, similar changes in self-reported ADL ability were not seen across groups neither when using the diagnosis specific instrument HAQ-Di, nor the generic ADL-Q. These findings are in correspondence with the results reported in a study by Amris et al. [17]. In that study, a 2-week multi-component rehabilitation course resulted in improvements in observed ADL ability in patients with chronic widespread pain, but these improvements were not reflected in scores of self-reported functional abilities on standardized questionnaires. Thus, our study supports the notion that observation-based evaluations of ADL ability may be more sensitive to measure changes following intervention than self-report.

The hypothesis that participants receiving hand exercise would have no increase in disease activity was confirmed. Thus, no negative effect on disease activity after exercise was seen either assessed locally by US examination, by count of swollen and tender joints of the hand, or globally as measured by inflammatory blood markers. In contrast, a significant increase in ESR was seen in the CIP_CONTROL_ group; however, ESR is a very slow reacting marker of disease activity and none of the other markers of disease activity showed the same tendency.

In this randomized trial, the exercise program did not reduce hand-related pain. In other studies, investigating the effect of hand-exercise programs in RA, there are diverse findings regarding reduction in pain after exercise; in two studies, reductions in pain measured on a VAS scale was reported [5, 41], whereas two other studies showed no pain reduction [6, 10]. The studies reporting pain reduction did not evaluate functional ability [5, 41]. The two studies reporting no pain reduction evaluated and reported improvements in functional ability (i.e., hand function, ADLs, pain, work performance, esthetics, and patient satisfaction with hand function) [8–10] and in evaluation of grip ability [6]. These findings suggest that pain and functioning are not necessarily closely linked factors, which is also seen in a study investigating exercise therapy in patients with impaired shoulder function [42]. Thus, the absence of any significant effect of our hand-exercise intervention on ability cannot be explained by unchanged pain in the hand after exercise.

One limitation of the study was not reaching the intended sample size. Still, the risk of overlooking a real group difference in primary outcome is minor, as both groups obtained a statistically significant increase in ADL motor ability with no indication of a group difference. Another limitation is that the disease duration was longer in the CIP_CONTROL_ compared to that in the CIP_XERCISE_ despite the randomized design. However, no pronounced difference in any of the functional measures, grip strength or disease activity score (DAS28) was seen between the two groups strongly indicating the groups were comparable.

## Conclusion

In conclusion, participants in both CIP_CONTROL_ and CIP_EXERCISE_ improved their ADL motor ability, but no statistically significant difference between groups was seen. Moreover, no differences between groups were seen in secondary outcomes. Thus, based on our results, it cannot be concluded that hand exercise as an add on to compensatory intervention further improves observed ADL ability in persons with RA-related impaired hand function.

## Additional file

Additional file 1: **Table S1.** Changes from baseline in primary and secondary outcomes analyses based on the intention-to-treat-population adjusted for baseline value, disease duration, and hand pain at baseline. (DOCX 20 kb)

Additional file 2: Patient handout exercise program. (PDF 1.48 kb)

Additional file 3: Detailed description of the hand-exercise programme. (PDF 386 kb)
