# Supplementary material for: Hand exercise for women with rheumatoid arthritis and decreased hand function: an exploratory randomized controlled trial
Source: Arthritis Res Ther. 2019 Jun 26;21:158. doi: 10.1186/s13075-019-1924-9 (PMC6595578; doi:10.1186/s13075-019-1924-9)
Supplement: Supplementary file 1 — Table S1. Changes from baseline in primary and secondary outcomes analyses based on the intention-to-treat-population adjusted for baseline value, disease duration, and hand pain at baseline. (DOCX 20 kb) [file 13075_2019_1924_MOESM1_ESM.docx]

Supplementarey table: Changes from Baseline in Primary and Secondary Outcomes Analyses based on the Intention-To-Treat-Population adjusted for baseline value, disease duration and hand pain at baseline.

|  | CIP_EXERCISE_ (n=27)  Mean change  (95% CI) | CIP_CONTROL_ (n=27 )  Mean change  (95% CI) | Group difference  Mean change (95%CI) | P-value |
| --- | --- | --- | --- | --- |
| AMPS* ADL motor ability | 0.31 (0.13 to 0.48) | 0.20 (0.05 to 0.35) | 0.11 (-0.14 to 0.55) | 0.38 |
| AMPS* ADL process ability | 0.08 (-0.02 to 0.19) | 0.04 (-0.06 to 0.14) | 0.04 (-0.11 to 0.20) | 0.57 |
| ADL-Questionnaire (ADL-Q) | 0.12 (-0.02 to 0.27) | 0.09 (-0.04 to 0.22) | 0.04 (-0.16 to 0.24) | 0.69 |
| HAQ-DI** | -0.13 (-0.26 to 0.02) | 0.3 (-0.08 to 0.13) | -0.16 (-0.33 to 0.01) | 0.06 |
| Hand strength and pain |  |  |  |  |
| Max grip strength (Kg)  Right  Left | 1.88 (0.51 to 3.25)  1.43 (-0.36 to 3.23) | 0.10 (-1.13 to 1.33)  -0.26 (-1.84 to 1.31) | 1.78 (-0.15 to 3.71)  1.70 (-0.80 to 4.21) | 0.07  0.18 |
| Hand pain, Activity (mm VAS  Right  Left | -3.46 (-11.83 to 4.91)  -6.63 (-15.37 to 2.12) | 1.17 (-6.25 to 8.58)  -3.02 (-4.72 to 10.76) | -4.62 (-16.29 to 7.05)  -9.65 (-21.84 to 2.55) | 0.43  0.12 |
| Hand pain, Rest (mm VAS)  Right  Left | -0.85 (-10.01 to 8.31)  -3.91 (-12.84 to 5.02) | 2.44 (-5.68 to 10.55)  4.25 (-3.66 to 12.16) | -3.28 (-16.06 to 9.49)  -8.16 (-20.61 to 4.29) | 0.61  0.19 |
| PainKillers (Number per day) | 0.03 (-0.21 to 0.15) | 0.02 (-0.18 to 0.15) | 0.01 (-0.27 to 0.24) | 0.92 |
| DAS28*** | -0.35 (-0.70 to 0.00) | 0.31 (-0.02 to 0.64) | -0.66 (-1.16 to -1.15) | 0.01 |
| Tender joint count | -0.82 (-2.35 to 0.72) | 0.65 (-0.71 to 2.01) | -1.47 (-3.62 to 0.68) | 0.17 |
| Swollen joint count | 0.00 (-0.57 to 0.57) | 0.36 (-0.14 to 0.86) | 0.36 (-1.16 to 0.43) | 0.36 |
| CRP^#^ | -0.99 (-3.16 to 1.18) | 1.80 (-0.31 to 3.91) | -2.79 (-5.95 to 0.37) | 0.08 |
| Disease activity (VAS) | -8.32 (-18.807 to 1.43) | -2.41 (-10.61 to 5.79) | -5.90 (-19.53 to 7.71) | 0.38 |
| ESR^##^ | -0.75 (-3.39 to 1.90) | 1.95 (-0.36 to 4.27) | -2.70 (-6.40 to 1.00) | 0.15 |
| Ultra Sound (US) |  |  |  |  |
| US score: synovial hypertrophy | 0.18 (-1.96 to 2.33) | 2.70 (-0.85 to 4.55) | -2.51 (-5.48 to 0.45) | 0.09 |
| Synovial perfusion (Doppler) | -0.28 (-1.79 to 2.34) | 2.27 (-0.49 to 4.04) | -1.99 (-4.84 to 0.86) | 0.17 |
| UL score total | -0.30 (-4.27 to 3.68) | 4.67 (1.24 to 8.09) | -4.96 (-10.45 to 0.53) | 0.08 |

*AMPS = Assessment of Motor and Process Skills; **HAQ-DI = Stanford Health Assessment Questionnaire Disability Index; *** DAS28 = Disease Activity Score 28; ^#^CRP = C-reactive protein; ^##^ESR = Erythrocyte Sedimentation Rate; ^###^AntiCCP = anti Cyclic Citrullinated Peptides.
