# Supplementary material for: Hand exercise for women with rheumatoid arthritis and decreased hand function: an exploratory randomized controlled trial
Source: Arthritis Res Ther. 2019 Jun 26;21:158. doi: 10.1186/s13075-019-1924-9 (PMC6595578; doi:10.1186/s13075-019-1924-9)
Supplement: Supplementary file 2 — Patient handout exercise program. (PDF 1.48 kb) [file 13075_2019_1924_MOESM2_ESM.pdf]

REGION H
Frederiksberg Hospital
Parker instituttet

Håndfunktionstræning til leddegigtpatienter

Opvarmning			
A	Knyt hånden led for led	Bøj først de yderste fingerled og slut med knyttet hånd.	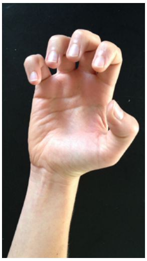 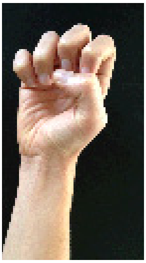 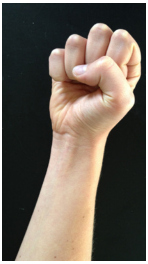
B	”Fingergang”	Læg håndfladen på bordet. Flyt fingrene enkeltvis i ’gang’ i tommelfingerens retning. Start med tommelfingeren.	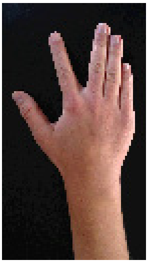 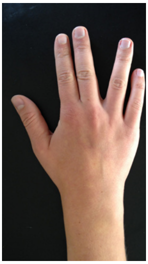
C	Fingerspredning	Hold håndfladen i bordet. Spred fingrene så meget som muligt og saml dem igen.	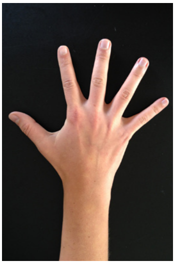
D	Tommeløvelse	Tommelfingeren føres skiftevis til de øvrige fingerspidser. ’Frem og tilbage’.	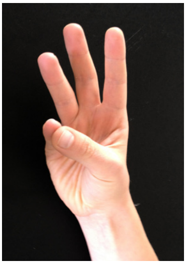

E	Håndledsvink	Hold håndfladen i bordet. Før håndfladen mod tommelfingerens side. Bevægelsen sker i håndleddet.	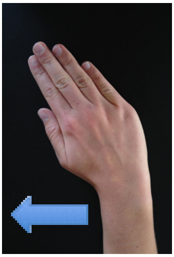
F	Håndledsrulning	Rul håndleddene rundt i begge retninger.	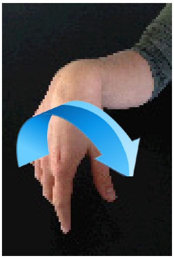
G	Skulderrulning	Rul skuldrene rundt.	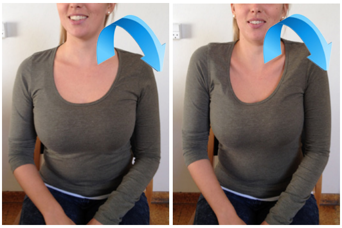
H	Hænder til nakke/lænd	Håndfladerne føres skiftevis til nakken og til lænden.	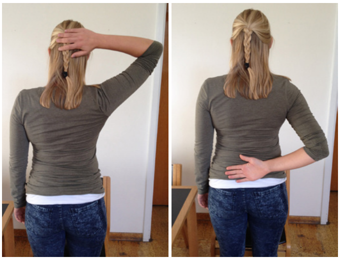

2. Styrketræning

Niveau 1 3x8 gentagelser. Borg 3-4 (Se bagside)
Niveau 2 3x8 gentagelser. Borg 4-5 (Se bagside)
Niveau 3 3x8 gentagelser. Borg 5-6 (Se bagside)
Niveau 4 3x10 gentagelser. Borg 5-6 (Se bagside)

Fingre

I	Stort greb	Voksen klemmes i hele hånden.	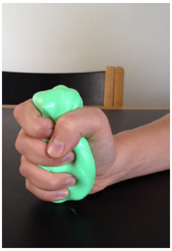
J	Pincetgreb	Tommel-, pege- og langefinger klemmer voksen.	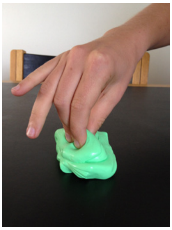
K	Fingersamling	En mindre klump voks klemmes mellem fingrene. Gentages mellem pege-, lange-, ring-, og lillefinger.	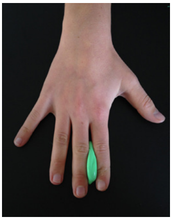

Håndled			
L		Den korte træningselastik sættes om hænderne, så den sidder over knoerne. Underarmene placeres på bordet med tommelfingeren opad. Der trænes én hånd ad gangen. Træk i elastikken ved at vippe håndleddet udad. Bevægelsen sker i håndleddet.	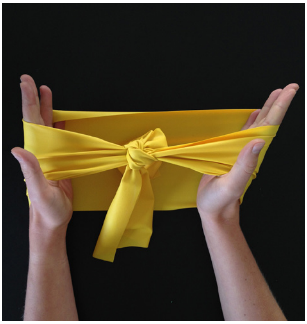
M		Håndfladen placeres under bordet. Der presses op mod undersiden af bordpladen. Hold i 5 sekunde	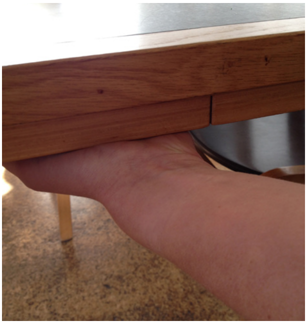

Albue

N	Biceps	Sid med den lange træningselastik under fødderne. Elastikkens ender vikles rundt om hænderne så elastikken ikke 'hænger'. Overkroppen holdes ret. Albuerne holdes ind mod siden. Armene trænes en ad gangen ved at bøje og strække i albueleddet. Idet der bøjes, føles elastikkens modstand.	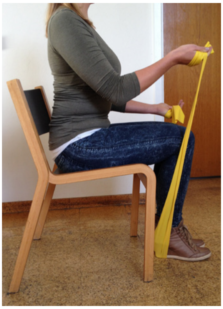 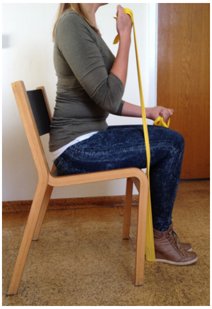
O	Triceps	Elastikken placeres som ved N, her skal du dog bøje dig let fremover. Armene trænes en ad gangen ved at strække i albueleddet og føre armen bagud. Idet der strækkes, føles elastikkens modstand.	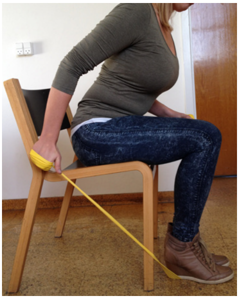

3. Nedvarmning

A	Knyt hånden led for led	Bøj først de yderste fingerled og slut med en knyttet hånd.	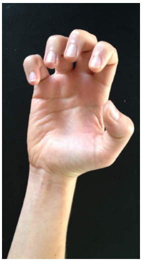 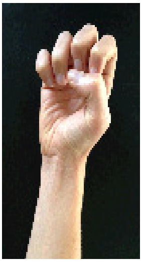 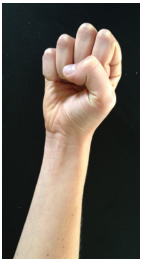
D	Tommel op-ponens	Tommelfingeren føres skiftevis til de øvrige fingerspidser. 'Frem og tilbage'.	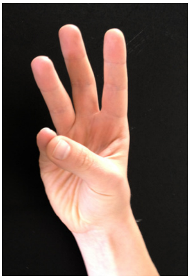
F	Håndledscir-kumduktion	Rul håndleddene rundt i begge retninger.	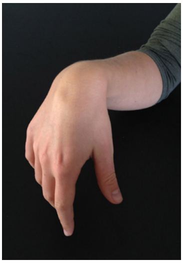
G	Skulderrul-ning	Rul skuldrene rundt.	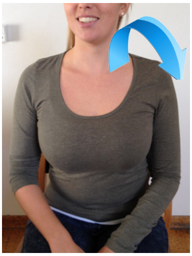 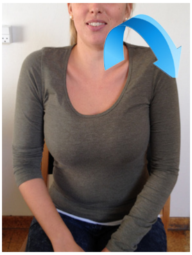

Håndfunktionstræning til leddegigtpatienter

Borg skala	
0	Ingenting
1	Meget let
2	Let
3	Moderat
4	Lidt hårdt
5	Hårdt
6	
7	Meget hårdt
8	
9	

Træningsniveau		
Niveau 1 3x8 gentagelser	Borg 3-4	Uge 1-2
Niveau 2 3x8 gentagelser	Borg 4-5	Uge 3-4
Niveau 3 3x8 gentagelser	Borg 5-6	Uge 5-6
Niveau 4 3x10 gentagelser	Borg 5-6	Uge 7-8
Rød elastik: Let<br>Grøn elastik: Tung		
