# Supplementary material for: Hand exercise for women with rheumatoid arthritis and decreased hand function: an exploratory randomized controlled trial
Source: Arthritis Res Ther. 2019 Jun 26;21:158. doi: 10.1186/s13075-019-1924-9 (PMC6595578; doi:10.1186/s13075-019-1924-9)
Supplement: Supplementary file 3 — Detailed description of the hand-exercise programme. (PDF 386 kb) [file 13075_2019_1924_MOESM3_ESM.pdf]

AN EXERCISES PROGRAM FOR THE HAND AND UPPER EXTREMITIES IN PATIENTS WITH RHEUMATOID ARTHRITIS

This exercise program runs for eight weeks; one weekly session with a physical therapist and 3-7 home exercise sessions. Each session with the physical therapist takes place in a closed room with a table and two chairs, lasting approximately 35 minutes. Each home exercise sessions lasted approximately 35 minutes. This exercise program consists of a warm-up period, a strength training period and a cool down period.

The focus of this exercise program is activation of the 'correct' muscles to optimize movement patterns of the hand and improve ROM. No resistance is used during the warm-up and cool down period, as the aim is to improving ROM and specifically for the warm-up exercises to preparing the joint for the strength training part. Each exercise in the strength training section is to be performed with good quality (assessed by the physical therapy) and resistance is provided by rubber bands or Theraputty.

BORG SCALE

The Borg scale (figure 1) is used to assess patients' self-reported experience of load. The scale is used during the strengthening exercises to assess if the resistance in the weakest hand is at the required level. A resistance level of 3-4 on the Borg scale is sought in the first period of the exercise program to minimize risk of flare-up. During the exercise period the patients' self-reported experience of load should increase to 7-8 Borg. Resistance should only be increased if the joint accept this (see below).

0	Nothing at all
1	Very easy
2	Easy
3	Moderate
4	Somewhat hard
5	Hard
6	
7	Very hard
8	
9	
10	Very, very hard

Figure 1. Borg's CR-10 rating of perceived exertion (RPA) scale from Borg GA. Psychophysical bases of perceived exertion. Med Sci Sports Exerc

PAIN

Pain is rate by the patient before, during and after each exercise session with the physical therapist on a numeric rating scale (NRS) with 0 = no pain and 10 = worst imaginable pain, to assess how the exercises affect the individual. Pain during the strengthening exercises is allowed if the pain subsides after each set. During the following 24 hours the patients should not experience an increase in pain. If such increase occurs, this might indicate that the load is too high and the exercises should be adjusted. The patient is instructed in how to rate and adjust load accordingly.

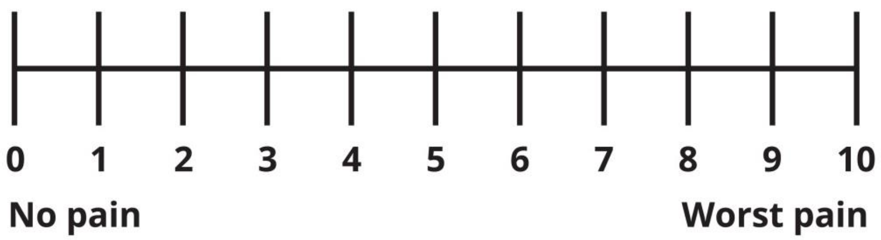

Figure 2. The numeric pain rating (NRS) scale used to assess patient pain intensity in the hand before, during and after each exercise.

THE EXERCISE PROGRAM

WARM-UP

The warm-up period consist of eight different exercises with the overall aim of preparing the joints for the strength exercises and to improve flexibility by targeting joint motions in the upper extremities known to be restricted in patients with RA.

Each exercise is to be performed 3 sets of 8 repetitions.

A: Knout the hand in three stages

Purpose: to increase mobility in the DIP, PIP and MIP joint of the hand by doing slow (app. 3 sec per rep.) controlled flexion of each joint.

Description: sitting with the arms resting on a table at elbow level with the palms turned upwards slowly bent each joint until a fist is formed. Strength all joint and repeated once more until full dosage has been achieved.

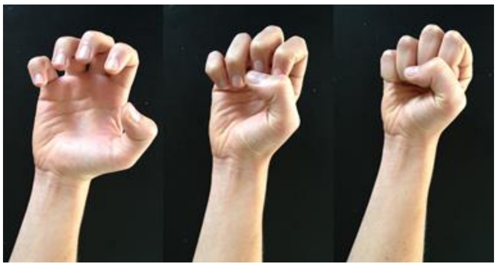

B: "walk" the finger 2 to 5 against the first finger one by one with the palmar side of the hand lying on a table

Purpose: to increase mobility in the abduction and adduction of the MIP joint by performing controlled abduction and adduction movement for each individual MIP joint.

Description: Place the palm on a flat surface, levelled with the elbow. Move the thumb as far away from the hand as possible (abduction), then move the rest of the fingers one at a time towards the thumb. When all fingers are as fare to one side as possible they are moved back one finger at the time starting with the little finger.

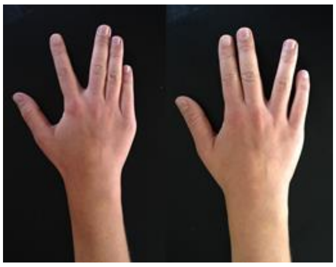

C: Spread the finger with the palmar side of the hand lying on a table

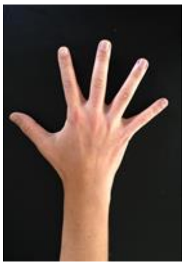

Purpose: to increase mobility in the abduction and adduction movement of the MIP joint by performing controlled abduction and adduction movement with all fingers.

Description: Place the palm on a flat surface, levelled with the elbow. Spread all fingers out at the same time and draw then together again.

D: Put the tip of the first finger to the tip of the other 4 finger one by one

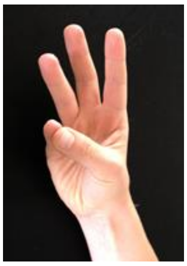

Purpose: To improve mobility and coordination in a flexion pattern.

Description: Open your hand and lead thumb meet the little finger so to make a circle, repeat with the thumb and 4. finger, then thumb and middle finger and last thumb and the index finger. Remember to make a circle each time.

E: Bend the stretched hand from side to side

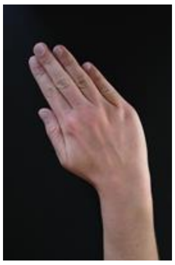

Purpose: to increase mobility in adduction and abduction in the wrist

Description: place your palm and lower arm on a flat surface levelled with the elbow. Without moving the lower arm, bent the wrist to the left and then right, use approximately 2. Seconds to complete the movement.

F: Make circles in the wrist joint

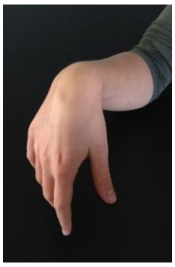

Purpose: To maintain and improve mobility in the wrist during rotation of radius.

Description: The lower arm should be free of any support, now rotate the wrist around, change direction regularly.

G: Make circles with the shoulders

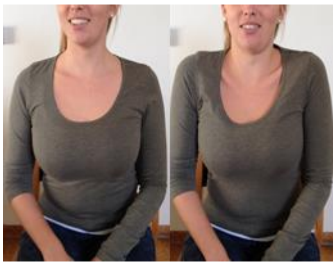

Purpose: To increase blood flow in the neck muscles and arms, loosen the muscle, and increase mobility of the shoulder and neck.

Description: Sit in a chair, with the back free of the chair and your hands placed in your lap. Look straight ahead and then lift your shoulders back, up and forward in a circle motion, after 8 repetitions go the other way around by starting with protraction of the shoulder than lift and then retraction.

H: Put alternating from back of the head and the loin

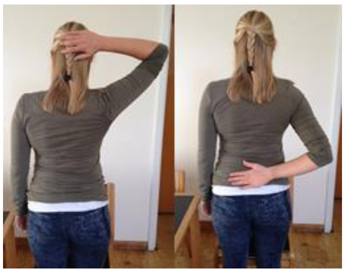

Purpose: To maintain or increase shoulder mobility related to common daily activities such as brushing your hair or putting on a coat.

Description: Sit in a chair with the back free of the chair, move one arm up and place the palm of your hand on the back of the head. The other arm is moved to the back and the back of the hand is placed at the loin. Simulations (If possible) shift the arms so the one that was placed on the loin now is at the back of the head and vice versa. Remember to keep the back straight. This exercise can also be done standing if this is deemed convenient.

STRENGTH EXERCISES

The first three exercises focus on the muscle in the lower arm and hand, a Theraputty is used to add resistance. The Theraputty will be with low resistance during the first sessions and then replaced to moderate or hard when the patient is ready; If the theraputty with the low resistance is difficult for the patient to use (increase pain levels) decrease the number of set or repetitions until completion without increase in pain is possible.

Exercise 4, 6, 7, and 8 are performed with a rubber band as resistance. Three bands are available (low, moderate or hard). The patient starts with the low resistance rubber band; if this is too hard (increases pain) decrease the number of set or repetitions until completion of the exercise without increase in pain is possible.

Exercise number 5 is performed with resistance from a table.

Each exercise is assessed with the Borg scale (see above, figure 1), and the exercise are progresses according to figure 3 during the 8 weeks assuming the patient reported pain remains the same or decreases.

Training intensity		
**Level 1** 3x8 repetitions	Borg 3-4	Week 1-2
**Level 2** 3x8 repetitions	Borg 4-5	Week 3-4
**Level 3** 3x8 repetitions	Borg 5-6	Week 5-6
**Level 4** 3x10 repetitions	Borg 5-6	Week 7-8

Figure 3. Training intensity during the 8 week intervention period.

I: Gross grip

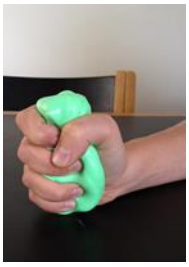

Purpose: To increase grip strength.

Description: Form the theraputty as a ball and place it in the palm of your hand; now flex all fingers simultaneous and hold for 2-3 seconds. Then release the grip, reassemble the theraputty into a ball and repeat until desired sets and repetitions are reached.

J: Finger pinch

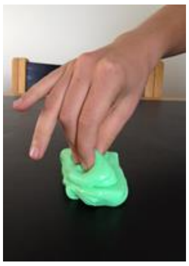

Purpose: To improve strength of the tweezer grip.

Description: Place the theraputting on a table, pinch thumb, index and middle finger together in a flexion patter for 2-3 seconds. Then release and repeat once more until desired sets and repetitions are completed. Then perform with the other hand. Remember to flex all joints in the three fingers during the exercise.

K: Finger adduction

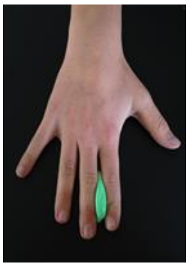

Purpose: To increase strength in the adductor muscles to improve strength.

Description: Make a ball of the theraputting (the size of a table-tennis ball) and place it between the index and middle finger. Place your hand on a table and squeezed the middle and index finger together around the theraputting for 2 seconds. Release move the theraputting to the middle and fourth finger and repeat the squeezed. Finish with a squeezed of the theraputting between the little finger and fourth finger. Repeat until the desired number of sets and repetitions has been reached.

L: Wrist extension

Purpose: To increase the strength of the wrist extensor muscles.

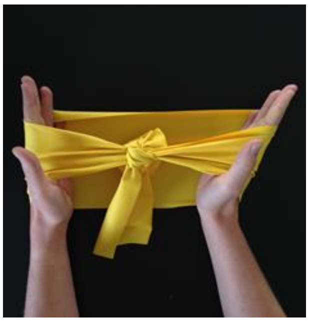

Description: Place the forearm at a horizontal level and elbow into the waist. Wrap the rubber band around both hands and tighten until there is tension when the hands are approximately 30 centimetres apart. While Holding this position the wrist is extended on wrist at a time until desired numbers of sets and repetitions have been reached.

M: Wrist flexions

Purpose: To increase the static strength during a small flexion position of the wrist.

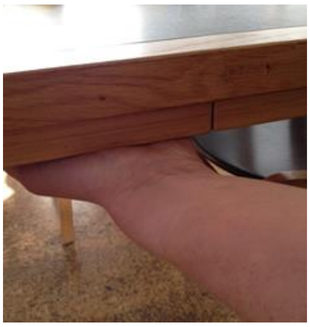

Description: Find a heavy table with a smoot surface underneath. Sit in a chair with the hands placed under the table. Lift the hands up and try to lift the table, hold for 5 seconds. Remember to keep the back straight and elbow at the waist to decrease stress on the shoulder joint.

N: Biceps

Purpose: Increase the strength of the biceps muscle of the upper arm.

Description: sit in a chair with the back free. Place both feet in the middle of the rubber band end wrap each end around the hands. Sit with a straight back and shorten the rubber band until there is tension when the hands are besides the knees. Keep the elbows fixed to the waist and flex in the elbow joint until the palm of the hand reached the shoulder. You can do it with one hand or both at the same time. This exercise can also be done standing if desired.

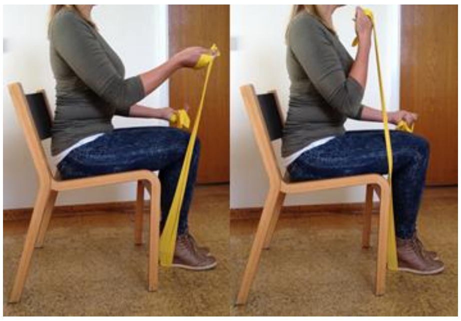

O: Triceps

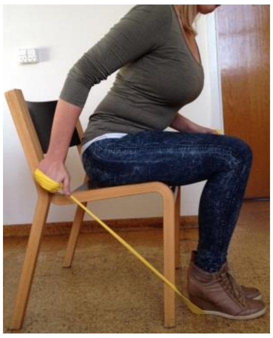

Purpose: To increase strength in the triceps muscle of the upper arm.

Description: Sit in a chair, as fare out on the edge as possible. Place your feet in the middle of the rubber band and wrap each end of the rubber band around the hands. Straighten the back and bend forward, hold the back straight until 45^0 flexion of the hip is reached (if possible); stay in this position during each set. Let your arm fall to the ground and tighten the rubber band until there is a small tension. Pull the elbow joint to the waist, then extent the elbow joint and move the arm forward again. This is repeated with one arm at a time until the desired sets and repetitions are reached. Remember to rest the back between sets. This exercise can be performed in a standing position by placing one foot in front of the other. The foot in front stands on the middle of the rubber band and each end is wrapped around the hands. Slightly bend the knee to get a stable stand and straighten your back and ben in the hip joint until a 45^0 flexion of the hip joint, keep this position during each set. Stretch the arms and tighten the rubber band. Move the elbow to the waist and then straighten the elbow joint to full extension.

COOL-DOWN

Exercise A, D, F, and G from the warm-up part are repeated with the same number of sets and repetitions as in the beginning.

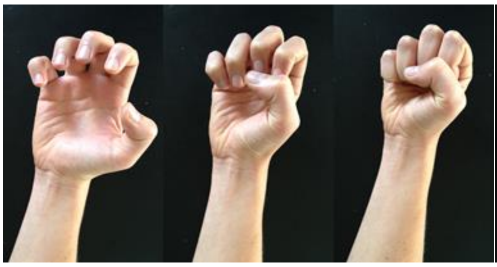
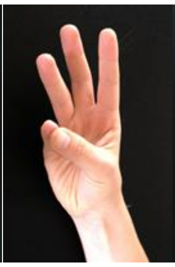
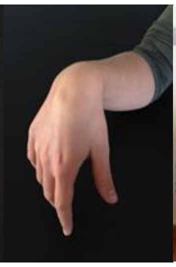
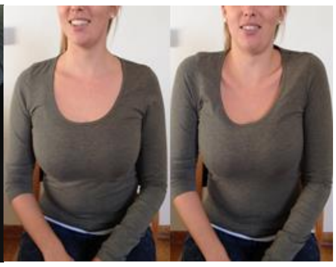

Exercise A **Exercise D** **Exercise F** **Exercise G**

RESCUE PROGRAM

If a patient experience a flare-up of symptoms that cannot be reduced by changed resistance, sets or number of repetitions a rescue program is provided to maintain the patient in the exercise pattern, without adding additional stress to the affected joint.

The rescue program contains the warm-up and cool down exercises and they are to be performed with the number of sets and repetitions as described above.
